# Trapping tsetse flies on water

**DOI:** 10.1051/parasite/2011182141

**Published:** 2011-05-15

**Authors:** C. Laveissière, M. Camara, J.B. Rayaisse, E. Salou, M. Kagbadouno, P. Solano

**Affiliations:** 1 380, route de la Virvée 33240 Saint-Romain-la-Virvée France; 2 PNLTHA Conakry Guinée; 3 CIRDES Bobo-Dioulasso 01 BP 454 Burkina Faso; 4 IRD, UMR IRD-CIRAD Trypanosomes, CIRDES Bobo-Dioulasso 01 BP 454 Burkina Faso

**Keywords:** tsetse, floating trap, mangrove, savannah, trypanosomose, West Africa, tsé-tsé, piège flottant, mangrove, savane, trypanosomose, Afrique de l’Ouest

## Abstract

Riverine tsetse flies such as *Glossina palpalis gambiensis* and *G. tachinoides* are the vectors of human and animal trypanosomoses in West Africa. Despite intimate links between tsetse and water, to our knowledge there has never been any attempt to design trapping devices that would catch tsetse on water. In mangrove (Guinea) one challenging issue is the tide, because height above the ground for a trap is a key factor affecting tsetse catches. The trap was mounted on the remains of an old wooden dugout, and attached with rope to nearby branches, thereby allowing it to rise and fall with the tide. Catches showed a very high density of 93.9 flies/”water-trap”/day, which was significantly higher (p < 0.05) than all the catches from other habitats where the classical trap had been used. In savannah, on the Comoe river of South Burkina Faso, the biconical trap was mounted on a small wooden raft anchored to a stone, and catches were compared with the classical biconical trap put on the shores. *G. p. gambiensis* and *G. tachinoides* densities were not significantly different from those from the classical biconical one. The adaptations described here have allowed to efficiently catch tsetse on the water, which to our knowledge is reported here for the first time. This represents a great progress and opens new opportunities to undertake studies on the vectors of trypanosomoses in mangrove areas of Guinea, which are currently the areas showing the highest prevalences of sleeping sickness in West Africa. It also has huge potential for tsetse control using insecticide impregnated traps in savannah areas where traps become less efficient in rainy season. The Guinean National control programme has already expressed its willingness to use such modified traps in its control campaigns in Guinea, as has the national PATTEC programme in Burkina Faso during rainy season.

## Abstract (fr)

Les tsé-tsé du groupe palpalis, en particulier les taxons riverains *Glossina palpalis gambiensis* et *G. tachinoides*, sont les vecteurs majeurs des trypanosomoses humaine (maladie du sommeil) et animales (nagana) en Afrique de l’Ouest. Malgré l’intimité des liens entre ces espèces et les formations aquatiques qui constituent leur habitat, il n’existe jusqu’à présent aucun moyen de piéger ces tsé-tsé sur l’eau. En zone de mangrove de Guinée, une difficulté supplémentaire est l’existence de la marée, qui affecte donc la hauteur à laquelle est placé le piège, facteur-clé de succès du piégeage. Le piège a donc été monté sur un morceau de bois flottant et attaché aux branches des palétuviers, suivant ainsi le niveau des marées à hauteur constante. Les captures ont montré que ce piège flottant capturait significativement plus de tsé-tsé (93,5 tsé-tsé par piège et par jour) que les pièges classiques sur terre. En zone de savane au Burkina Faso, sur la rivière Comoé, le piège biconique a été monté sur un petit radeau de bois et immobilisé au moyen d’une ancre. Les comparaisons avec des pièges biconiques posés sur la rive n’ont pas montré de différence significative pour les deux espèces *G. p. gambiensis* et *G. tachinoides*. La mise au point de ces pièges flottants ouvre de nouvelles perspectives sur l’étude de ces vecteurs en zone de mangrove, où peu voire pas de données bio-écologiques existent alors que les prévalences de la trypanosomiase humaine africaine (THA) sont fortes. Cet outil a aussi un fort potentiel d’application pour la lutte anti-tsé-tsé en zone de savane en saison des pluies, lorsque les pièges ou écrans classiques ne sont plus efficaces. Le Programme national de lutte contre la THA en Guinée et le projet PATTEC au Burkina Faso ont manifesté leur intention d’utiliser ces pièges flottants dans leurs campagnes de lutte.

## Introduction

Tsetse flies are the vector of human and animal Trypanosomoses that still cause thousands of lethal cases to humans (Simarro *et al.*, 2008), and millions dollars of losses for agriculture and livestock (Budd *et al.*, 1999). In West Africa, the area currently the most prevalent for sleeping sickness is the mangrove area of Guinea (Camara *et al.*, 2005), whereas in savannahs the main problem is animal trypanosomosis (Courtin *et al.*, 2008). The main vector of both human and animal African trypanosomiasis (HAT and AAT) in West Africa is *Glossina palpalis gambiensis*, which lives from the savannah to the mangrove. This tsetse is a riverine species since it is intimately linked to water: it disperses mainly along rivers and forest vegetation, and can hardly go out from this habitat due to its hygrometrical needs. Riverine forests correspond to its resting and reproductive sites (Challier, 1973). Larviposition in savannah usually takes place on river shores, whereas it remains unknown in mangrove. Quite unexpectedly, despite these intimate links between tsetse and water, to our knowledge there has never been any attempt to design trapping devices that would catch tsetse on water.

One of the most efficient strategies to break the trypanosomosis cycle is to control the vector. This can be done by using insecticide impregnated traps and targets, which is a very efficient technique, environmentally friendly, cheap, and doable by local communities (Laveissière & Penchenier, 2005). These traps are also the most used devices to sample alive tsetse: in this case they are used without insecticides, using a cage on top of the trap that will allow the collection of the flies. Two of them are mainly used in West Africa: the biconical Challier-Laveissière trap (Challier *et al.*, 1977), and its simplified derivative, the monoconical Vavoua trap (Laveissière & Grébaut, 1990). These traps are usually placed along the river shores in savannah, or in places frequented by humans near the water in forested regions.

However in mangrove, very few studies, if any, have been undertaken on tsetse ecology. This may be due to the fact that they inhabit this typical mangrove habitat, which is accessible only by boat. There was therefore a need to develop a trap that would be able to sample tsetse in their natural habitat, *i.e.* on the water. However in the mangrove habitat, the tide makes it impossible to put classical traps, since they would be either flooded by high tide, or be totally unefficient at low tide. Indeed the lower parts of the trap have to be placed at a maximum of 30 cm up the ground to efficiently catch tsetse, and height is known to significantly affect trapping efficiency (Laveissière et al., 1987).

Here we report the adaptation of the Vavoua and biconical traps to water habitat, respectively in mangrove in Guinea, and in savannah in Burkina Faso.

## Material and Methods

Different adaptations were implemented according to tsetse habitat before reaching the best compromise (data not shown).

### • Mangrove (Guinea)

**Fig. 1.**
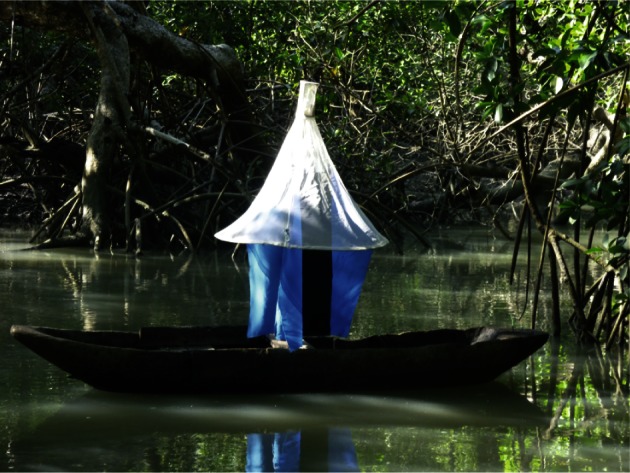
Floating Vavoua trap in the mangrove of the Dubreka focus, Guinea.

As it can be seen in Fig. 1, the trap was mounted on the remains of an old wooden dugout, and attached with rope to nearby branches thereby allowing it to rise and fall with the tide. Trap catches between the classical trap and the new one were compared in different habitats at the same time, in dry season. The water-adapted trap (here the Vavoua) was set in the mangrove channels, whereas the classical Vavoua traps had been sited in typical tsetse habitats (forest, encampments, villages, riverside docks, tracks, wells). A negative binomial regression was used to compare densities according to habitat, using the Stata Software (Stata Corporation).

### • Savannah (Burkina Faso)

**Fig. 2.**
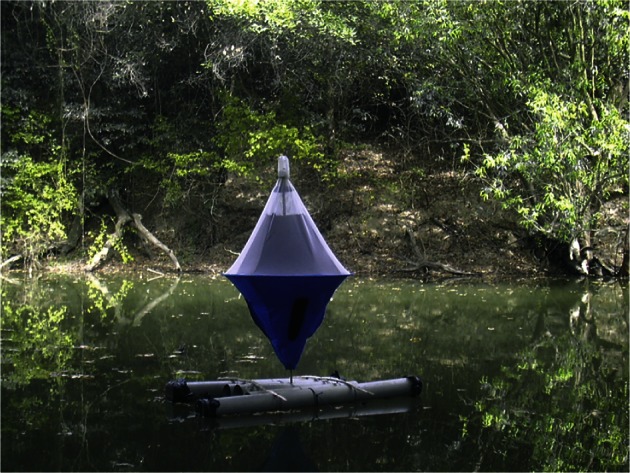
Floating biconical trap on the Comoe river, Burkina Faso.

The biconical trap was mounted on a small wooden raft anchored to a stone as illustrated in Fig. 2. Catches were compared between the water-adapted trap and the classical one at the same time (dry season also) in the same habitat. Traps were settled on the Comoe river in the area of Folonzo (south of Burkina Faso), and catches were compared following a 2*2 latin squares design repeated 12 times. Daily catches were normalized and variances homogenized using a log10 (n + 1) transformation. An ANOVA of these transformed means was done using the freely available Genstat Software. To provide a common index of the effect of trap type on catches, the detransformed mean catch of tsetse from the water-adapted trap was expressed as the proportion of that from the standard one and the value was termed the catch index (see also Omolo *et al.*, 2009; Rayaisse *et al.*, 2010). The difference between the two types of trap was significant when the p value was < 0.05.

## Results

### • Guinea

As it can be seen in Table I, catches showed a very high density of 93.9 flies/“water-trap”/day (f/t/d), which was significantly higher (p < 0.05) than all the catches from other habitats where the classical trap had been used, these latter ranging from 4 to 28 f/t/d. All tsetse caught belonged to *G. p. gambiensis*. Although this experimental design did not allow to know whether the modified trap caught more than a normal trap, or alternatively if tsetse were more present in mangrove channels than in other habitats, it is worthy of note that it was the only one possible, since no other trap than the modified one could be used in these channels.

**Table I.** *Glossina palpalis gambiensis* apparent density per biotope in mangrove habitat, Guinea.

Biotope	Apparent density	p
Mangrove channel (control)	93.8 ± 32.8	–
Dry channel	29 ± 16.8	0.000
Encampment	12.5 ± 4	0.000
River	14.5 ± 5.8	0.000
Riverside dock	25.75 ± 6.9	0.000
Forest	25.75 ± 3.3	0.000
Village	4.25 ± 1.5	0.000
Wells	8.5 ± 1.9	0.000
Track	10 ± 2.6	0.000

Apparent densities in all biotopes were compared to the density of the mangrove water channel by using a binomial regression (see text for details). All values were significantly lower than the density obtained in the mangrove channel.

### • Burkina Faso

In Table II are shown the results of the comparisons between the modified biconical and the classical one on the Comoe river, where two species were caught, *G. p. gambiensis* and *G. tachinoides*. *G. p. gambiensis* densities, although being slightly lower using the modified trap, were not significantly different than those from the classical biconical one. It was the same for *G. tachinoides*, this latter being caught in much higher densities.

**Table II.** Results of comparisons between the modified (floating) biconical trap and the classical one on the Comoe river in Burkina Faso.

Trap	Gpgm	Catch index	^G^Pg^f^	Catch index	Gpg	Catch index	Gtm	Catch index	Gtf	Catch index	Gt	Catch index
Classical	1.53	1	0.98	1	2.52	1	16.82	1	31.14	1	48.32	1
Floating	0.95	0.62	0.5	0.51	1.44	0.57	15.03	0.89	23.27	0.75	38.99	0.81
n	12		12		12		12		12		12	
p	0.33		0.25		0.25		0.55		0.19		0.28	
sed	0.11		0.1		0.13		0.07		0.09		0.08	

Densities are detransformed ones. Catch index is as described in the text. n = number of repeats (*i.e*. number of days of capture); p = probability; sed = standard deviation; Gpg = *G. p. gambiensis*; Gt = *G. tachinoides*; m = male; f = female.

## Discussion

The adaptations described here have allowed to efficiently catch tsetse on the water, which to our knowledge is reported here for the first time. This represents a great progress and opens new opportunities to undertake studies on the vectors of Trypanosomoses in mangrove areas of Guinea, which are currently the areas showing the highest prevalences of sleeping sickness in West Africa (Camara *et al.*, 2005; Simarro *et al.*, 2008). It also has huge potential for tsetse control using insecticide impregnated traps in savannah areas where traps become less efficient in rainy season, either because they are destroyed by floods, or because grass is too high and traps are no longer visible to tsetse. The adaptation described here makes it possible to still have an impact on tsetse populations during the rainy season. Should the efficiency of this modified trap had been lower than the classically used ones, it would still be very useful since the classical ones can not be used at all during the rainy season in savannah, nor they can be used at any season in the mangrove habitat. The Guinean National control programme has already expressed its willingness to use such modified traps in their control campaigns in Guinea, as has the national PATTEC programme in Burkina Faso during rainy season (I. Sidibe, pers. com.).

This first improvement provides room for further improvements in trapping efficiency in these areas, for instance by modifying size and shape of these floating devices, as described in Lindh *et al.* (2009) for classical traps and targets. This modified trap could also be used wherever tsetse are intimately linked to water, which represent most of the cases for riverine species, such as *G. fuscipes* s.l. which is the main vector of sleeping sickness throughout Central Africa.

## References

[R1] Budd L. Economic analysis, *In*: DFID-funded tsetse and trypanosomiasis research and development since 1980. Department for International Development: Livestock Production Programme, Animal Health Programme/Natural Resources Systems Programme, Chatham, UK, 1999

[R2] Camara M., Kaba D., Kagbadouno M., Sanon J.R., Ouendeno F. & Solano P. La trypanosomose humaine africaine en zone de mangrove en Guinée caractéristiques épidémiologiques et cliniques de deux foyers voisins. Médecine Tropicale, 2005, 65, 155–161 16038356

[R3] Challier A. Écologie de *Glossina palpalis gambiensis* Vanderplank, 1949 (Diptera-Muscidae) en savane d’Afrique occidentale. ORSTOM, Paris, 1973

[R4] Challier A., Eyraud M., Lafaye A. & Laveissière C. Amélioration du rendement du piège biconique pour glossines (Diptera : Glossinidae) par l’emploi d’un cône inférieur bleu. Cahiers ORSTOM, série Entomologie Médicale et Parasitologie, 1977, 15, 283–286

[R5] Courtin F., Jamonneau V., Duvallet G., Garcia A., Coulibaly B., Cuny G. & Solano P. Sleeping sickness in West Africa (1906–2006): changes in spatial repartition and lessons from the past. Tropical Medicine and International Health, 2005, 13, 334–344 18397396 10.1111/j.1365-3156.2008.02007.x

[R6] Laveissière C. & Grébaut P. Recherches sur les pièges à glossines. Mise au point d’un modèle économique : le piège “Vavoua”. Tropical Medicine and Parasitology, 1990, 41, 185–192 2166330

[R7] Laveissière C. & Penchenier L. Manuel de lutte contre la maladie du sommeil. IRD Éditions, Paris, 2005, 366 p.

[R8] Laveissiere C., Couret D. & Grebaut P. Recherche sur les écrans pour la lutte contre les glossines en région forestière de Côte d’Ivoire Mise au point d’un nouvel écran. *Cahiers ORSTOM*, série Entomologie Médicale et Parasitologie, 1987, 25, 145–164

[R9] Lindh J.M., Torr S.J., Vale G. & Lehane M. Improving the cost-effectiveness of artificial visual baits for controlling the tsetse fly *Glossina fuscipes fuscipes*. Plos Neglected Tropical Diseases, 2009, 3 (7) : e474 19582138 10.1371/journal.pntd.0000474 PMC2699553

[R10] Omolo M.O., Hassanali A., Mpiana S., Esterhuizen J., Lindh J., Lehane M.J., Solano P., Rayaisse J.B., Vale G.A., Torr S.J. & Tirados I. Prospects for developing odour baits to control *Glossina fuscipes* spp., the major vector of human African trypanosomiasis. PLoS Neglected Tropical Diseases, 2009, 3 (5), e435 19434232 10.1371/journal.pntd.0000435 PMC2674566

[R11] Rayaisse J.B., Tirados I., Kaba D., Dewhirst S., Logan J., Diarrassouba A., Omolo M., Solano P., Lehane M.J., Pickett J., Torr S.J., Esterhuizen J. Prospects for odour bait development to control vectors of Trypanosomiasis in West Africa, the tsetse flies *Glossina tachinoides* and *G. palpalis* s.l. PLoS Neglected Tropical Diseases, 2010, 4, e632 20300513 10.1371/journal.pntd.0000632 PMC2838779

[R12] Simarro P.P., Jannin J. & Cattand P. Eliminating human African trypanosomiasis: where do we stand and what comes next. PLoS Medicine, 2008, 5, e55 18303943 10.1371/journal.pmed.0050055 PMC2253612

